# Enhanced Toxicity of Bisphenols Together with UV Filters in Water: Identification of Synergy and Antagonism in Three-Component Mixtures

**DOI:** 10.3390/molecules27103260

**Published:** 2022-05-19

**Authors:** Błażej Kudłak, Natalia Jatkowska, Wen Liu, Michael J. Williams, Damia Barcelo, Helgi B. Schiöth

**Affiliations:** 1 Department of Analytical Chemistry, Faculty of Chemistry, Gdańsk University of Technology, 11/12 Narutowicza Str., 80-233 Gdańsk, Poland; blakudla@pg.edu.pl; 2 Functional Pharmacology, Department of Neuroscience, Uppsala University, 751 24 Uppsala, Sweden; wen.liu@neuro.uu.se (W.L.); michael.williams@neuro.uu.se (M.J.W.); helgi.schioth@neuro.uu.se (H.B.S.); 3 Catalan Institute for Water Research (ICRA), Parc Científic i Tecnològic de la Universitat de Girona, C/Emili Grahit, 101 Edifici H2O, E-17003 Girona, Spain; dbcqam@cid.csic.es

**Keywords:** bisphenol A, bisphenol A analogues, sunscreens, toxicity of mixtures, acute toxicity, environmental pollution

## Abstract

Contaminants of emerging concern (CEC) localize in the biome in variable combinations of complex mixtures that are often environmentally persistent, bioaccumulate and biomagnify, prompting a need for extensive monitoring. Many cosmetics include UV filters that are listed as CECs, such as benzophenone derivatives (oxybenzone, OXYB), cinnamates (2-ethylhexyl 4-methoxycinnamate, EMC) and camphor derivatives (4-methylbenzylidene-camphor, 4MBC). Furthermore, in numerous water sources, these UV filters have been detected together with Bisphenols (BPs), which are commonly used in plastics and can be physiologically detrimental. We utilized bioluminescent bacteria (Microtox assay) to monitor these CEC mixtures at environmentally relevant doses, and performed the first systematic study involving three sunscreen components (OXYB, 4MBC and EMC) and three BPs (BPA, BPS or BPF). Moreover, a breast cell line and cell viability assay were employed to determine the possible effect of these mixtures on human cells. Toxicity modeling, with concentration addition (CA) and independent action (IA) approaches, was performed, followed by data interpretation using Model Deviation Ratio (MDR) evaluation. The results show that UV filter sunscreen constituents and BPs interact at environmentally relevant concentrations. Of notable interest, mixtures containing any pair of three BPs (e.g., BPA + BPS, BPA + BPF and BPS + BPF), together with one sunscreen component (OXYB, 4MBC or EMC), showed strong synergy or overadditive effects. On the other hand, mixtures containing two UV filters (any pair of OXYB, 4MBC and EMC) and one BP (BPA, BPS or BPF) had a strong propensity towards concentration dependent underestimation. The three-component mixtures of UV filters (4MBC, EMC and OXYB) acted in an antagonistic manner toward each other, which was confirmed using a human cell line model. This study is one of the most comprehensive involving sunscreen constituents and BPs in complex mixtures, and provides new insights into potentially important interactions between these compounds.

## 1. Introduction

For years, the increase in anthropopressure on the natural environment, resulting from economic, industrial and agricultural activity, has caused significant changes to both abiotic and biotic systems. Numerous reports provide information about contaminant facilitated degradation and disintegration of the natural environment. For several years, a key interest has been to understand the environmental and biological effects of compounds generally referred to as CECs (contaminants of emerging concern) [1]. CECs typically exhibit a high level of environmental persistence and are not easily biodegradable, often demonstrating the ability to bioaccumulate and biomagnify. Moreover, most of these contaminants are considered to be bioavailable and capable of disrupting endocrine systems [2]. 

The cosmetic industry is flooding the market with numerous products in different formats. A wide range of new cosmetics enter the market each season, including products that protect the skin from harmful UV radiation. Many of these products contain highly diverse chemicals, including UV filters, which are considered CECs. These chemicals encompass organic compounds that are classified into different families, such as benzophenone derivatives (e.g., oxybenzone (OXYB)), salicylates, cinnamates (e.g., 2-ethylhexyl 4-methoxycinnamate (EMC)), camphor derivatives (e.g., 4-methylbenzylidene-camphor (4MBC)), p-aminobenzoic acid and its derivatives. Furthermore, CEC listed inorganic compounds are also used as UV filters, such as titanium dioxide (TiO_2_) and zinc oxide (ZnO), which create a physical barrier to excessive solar radiation [3]. 

Oxybenzon (OXYB) is a popular ingredient in UV-protective products, owing to its low production price, ease of synthesis and high solubility in organics. Along with its threats to animal species, OXYB also threatens plant species, due to the fact that at relatively low concentrations (e.g., 5 µM), it can cause negative reactions in plant photosynthesis systems [4], excessive production of reactive oxygen species (ROS) and disassembly of membranes in algae [5]. Furthermore, several studies performed on fish (*Danio rerio* and *Oryzias latipes*) demonstrate that exposure to OXYB (at 749 µg/L median measured levels) greatly impacts fecundity, lowering the number of eggs laid and hatched [6].

2-Ethylhexyl 4-methoxycinnamate (EMC) is used as a UV filter to help in the treatment of sunburns and scars. EMC is detectable in samples of almost all everyday consumer products, in dust collected in private and public buildings, sewage sediments, surface waters and even mammalian excrements at levels of up to several hundred nM [7]. Considered a CEC, in some species it has been shown to be a possible factor in reproduction impairments at very low levels, including 0.4 mg/kg (*Potamopyrgus antipodarum*) and 10 mg/kg (*Melanoides tuberculata*) [8]. 

4-Methylbenzylidene-camphor (4MBC) is also commonly used in multi-ingredient sunscreen compositions. 4MBC is also detectable in dust and sewage waters at elevated levels (1780 µg/kg d.w.) [9], resulting from the flushing of household waters, surface outflows and industrial discharges [10]. Moreover, like other UV filters, 4MBC may be present in plastics and other everyday items, posing an additional risk to marine ecosystems.

Much more is known about the environmental impact of bisphenol analogues, including BPA, BPS and BPF. These compounds are commonly used in the production of polycarbonate plastics, epoxy resins, food storage containers, plastic helmets, toys, as well as many other products. BPF-based resins have very good viscous properties and are resistant to organic solvents; therefore, it is more and more commonly used in industry. Furthermore, public acceptance that BPA exposure induces hormonal impairment has led to a considerable increase in the use of BPS. Unfortunately, this means that “BPA-free” products are not free of BPS. The presence of bisphenol analogues is ubiquitous, having been reported in indoor dust, foodstuffs, surface water, sewage sludge, sediment samples, human urine, blood and in maternal and cord serum, generally with concentration levels lower than BPA, but in the same order of magnitude [11,12,13,14]. 

Toxicological studies have been carried out for most of the UV filter and bisphenol compounds, which revealed that they cause adverse changes in living organisms, including affecting survival, behavior, growth, metabolism, development and reproduction, in addition to showing hormonal-like activity [15]. Given the properties of such xenobiotics, it is essential to carry out research aimed at determining the ecological impact resulting from their common presence in the environment. Unfortunately, most research focuses on the quantification of contaminants in the samples. Such information is obviously very useful, yet it may not be sufficient for a complete assessment of the environmental condition, as it does not allow for the determination of an actual biological impact of given pollutant, especially when considering the fact that contaminants do not occur individually within the environment but are found as mixtures. 

The co-occurrence and the resulting interactions of contaminants make it extremely difficult to foresee the environmental and physiological effects of such exposure. Therefore, it becomes necessary to identify and determine the type of interactions that occur between contaminants (first in model mixtures then in relation to environmental concentration levels). To date, the research carried out in this area has primarily focused on determining the impact of mixtures comprising UV filters [16]. However, it is apparent in the environment that such contaminants co-occur in mixtures with other xenobiotic compounds, which can significantly alter the level and action of their toxicity. Our study is the first systematic attempt to understand the combined impact of these emerging pollutants using bioluminescent bacteria and then testing if these responses can be recapitulated in human cancer cells. In this way, we not only test the effects of various pollutant mixtures, but try to confirm the relevance of the results using human cells.

## 2. Results

In the following subsections, the results of mixture toxicity studies with bioluminescent bacteria are given.

### 2.1. Impact of Three-Component Mixtures on BPA Toxicity

#### 2.1.1. BPA + OXYB + Second Bisphenol

**Figure 1:**
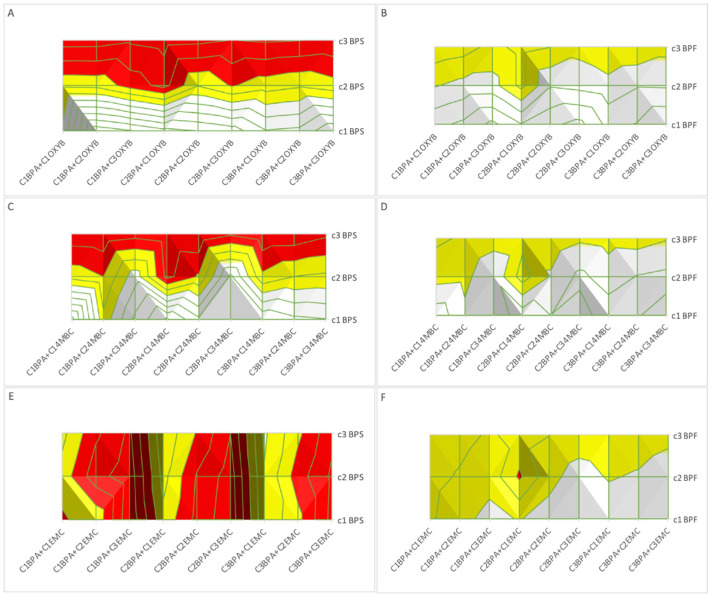
MDR values of *Aliivibrio fischeri* bioluminescent bacteria results for: (**A**) CA modeling of the BPA, OXYB and BPS mixture, (**B**) CA modeling of the BPA, OXYB and BPF mixture, (**C**) CA modeling of the BPA, 4MBC and BPS mixture, (**D**) CA modeling of the BPA, 4MBC and BPF mixture, (**E**) CA modeling of the BPA, EMC and BPS mixture, (**F**) CA modeling of the BPA, EMC and BPF mixture (*n* = 2). Red color indicates confirmed synergy, blue indicates antagonism, while yellow and green refer to under- and overestimation, respectively.

Results of the impact of a third component on the toxicity of BPA and OXYB—in the form of MDR values—are given in Supplementary Table S1 and Figure 1. With the CA model, the impact of BPS on the BPA + OXYB mixture had a clear concentration dependence trend (Figure 1A), whereby increasing BPS concentrations moved the mixture’s toxicological potential into strong synergy. On the other hand, in the IA model, at the lowest concentration of BPS, the impact clearly went in the direction of antagonism. A similar trend was observable when the third component was BPF, but the magnitude of this action was clearly weaker.

#### 2.1.2. BPA + OXYB + Second UV Filter

Underestimation was observed in nine cases in our studies to understand the impact of EMC on a BPA + OXYB mixture. At the lowest concentrations, this three-component mixture had a trend towards synergy (for CA modeling, refer to Supplementary Table S1 and Figure 1B). This may suggest and justify continuing studies in the direction of low-content mixtures, which more precisely reflect environmentally relevant mixture compositions. Nevertheless, both IA and CA models adequately showed variation of toxicity with three-component mixtures in our study with bioluminescent bacteria.

#### 2.1.3. BPA + 4MBC + Second Bisphenol Analogue

Results of a third component on the toxicity of a BPA and 4MBC mixture are presented in Supplementary Table S2 and Figure 1C, in the form of MDR values. Interestingly, a trend was observed when the concentration of a second bisphenol analogue (BPS) was increased, where the mixture components became synergistic in their behavior (such as in the case of BPA + OXYB + second bisphenol analogue). In the case of BPF, with CA modeling, this trend was also observable, but the magnitude was weaker. IA modeling resulted in a tendency towards overestimation.

#### 2.1.4. BPA + 4MBC + Second UV Filter

The impact of EMC on the toxicity of a BPA + 4MBC mixture was very well forecasted by the IA model (refer to Supplementary Table S2); The CA model predicted several instances of underestimation, and this behavior was observed in only six cases of EMC C1 and C2. 

#### 2.1.5. BPA + EMC + Second Bisphenol Analogue

A CA model of a BPA, EMC and BPS mixture showed synergistic effects in all cases and underestimated the impact (Figure 1E and electronic Supplementary Table S3). Some concentration dependence was also visible in mixtures containing the lowest BPS content, as well as with BPA C2 and C3. With increasing EMC concentration, the mixture became more synergistic. Similar behavior was noticeable for mixtures where BPF was present as the third component (Figure 1F), but there was only one confirmed case of synergy. On the other hand, both for BPS and BPF, in almost all cases, the IA model showed no significant interactions (only one case of overestimation was observed—C1 BPS + C1 BPA + C2 EMC).

### 2.2. Impact of Three-Component Mixtures on BPS Toxicity

#### 2.2.1. BPS + OXYB + Second Bisphenol

MDR results of the BPF impact on a BPS + OXYB mixture are presented in Supplementary Table S4 and Supplementary Figure S2A. For all cases, for BPS C1 no significant discrepancies between observed and calculated toxicity values were present using either the CA or the IA model. The OXYB and BPF mixture showed underestimation (for C1 BPF) and synergy (C2 and C3 of BPF) with BPS C2, having a clear concentration-dependence trend; interestingly, BPS C3 made all mixtures synergistic.

#### 2.2.2. BPS + OXYB + Second UV Filter

The impact of 4MBC and EMC on BPS + OXYB toxicity are presented in Supplementary Figure S2B,C, respectively, as well as in Supplementary Table S4. At the lowest BPS concentration studied, both models indicated no interactions between any chemical found in the mixture. The BPS C2 and C3 concentration dependence trend was similar to one observed with the BPS + OXYB + BPF cocktail; however, the IA model showed a trend towards overestimation. Interestingly, EMC C2 and C3 exhibited a strong undeniable synergistic impact on all BPS + OXYB mixtures, while the impact of EMC C1 was underestimated, with a trend towards synergy at the lowest concentrations of all analytes present in the cocktail. 

#### 2.2.3. BPS + 4MBC + Second UV Filter

Results of MDR for mixtures containing BPS, 4MBC and EMC are presented in Supplementary Figure S2D, as well as in Supplementary Table S5. EMS had a tendency to underestimate the impact on the BPS and 4MBC mixture in almost all cases studied. Of note, at environmentally relevant levels, almost all other mixtures confirmed the synergistic impact (with CA model) of these pollutant cocktails. 

#### 2.2.4. BPS + EMC + Second Bisphenol

The CA model of BPS + EMC mixtures with BPF again showed interesting trends (Supplementary Figure S2E and Supplementary Table S6). The lowest BPS concentrations, with increasing concentrations of BPF and EMC, had a trend towards synergy. The magnitude of this trend was not strong but was noticeable. The BPS C2 and C3 concentration levels trended towards a slight weakening of synergy, the only exception being the mixture of BPS C2 + EMC C3 + BPF C2, which showed signs of underestimation. The IA model was again resistant to concentration variations and was not suitable for predicting plausible synergy/antagonism of chemicals acting in a similar manner.

### 2.3. Impact of Three-Component Mixtures on BPF Toxicity

#### 2.3.1. BPF + 4MBC + Second Bisphenol

In the case of CA modeling, BPS had a clear synergistic impact on the BPF + 4MBC toxicological output of the bioluminescent bacteria. Interestingly, a trend of going from underestimation to synergy was clearly correlated with increasing BPS content (Supplementary Figure S3A and Supplementary Table S7). From an aqueous ecosystems point of view, this may demonstrate the detrimental effects of replacing BPA with BPS in everyday products/plastics production and uncontrolled waste disposal.

#### 2.3.2. BPF + 4MBC + Second UV Filter

The impact of EMC and OXYB on the toxicity of BPF + 4MBC is presented in a graphic manner in Supplementary Figure S3B,C, respectively (as well as in Supplementary Table S7). No cases of synergy were present (except one result of the mixture with OXYB), and underestimation was confirmed in all cases of BPF C3.

#### 2.3.3. BPF + OXYB + Second UV Filter

The results of MDR modeling for BPF + OXYB + EMC are presented in Figure S3D (and in Supplementary Table S8). The behavior of these three-component mixtures was similar to the ones shown in Supplementary Figure S2B,C, with many underestimated cases confirmed. Only one case of synergy was indicated. 

### 2.4. Mixture of Three UV Filters Studied

**Figure 2:**
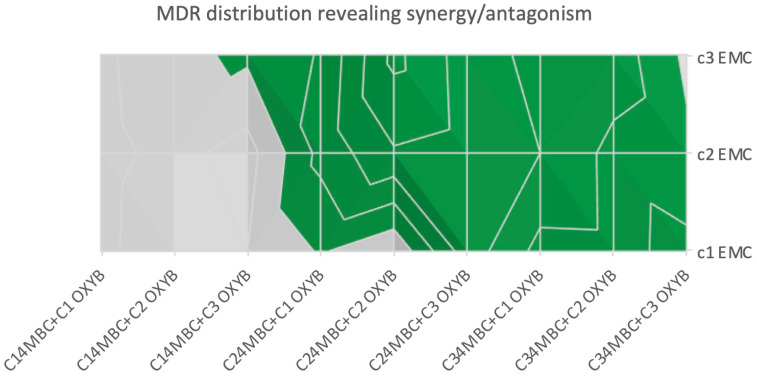
MDR values for IA modeling of the 4MBC, OXYB and EMC mixture of bioluminescent bacteria results (*n* = 2). Red color indicates confirmed synergy, blue indicates antagonism, while yellow and green refer to under- and overestimation, respectively.

The CA model for three compounds theoretically acting with the same MOA (Mode of Action) reflects the impact of such mixtures on bioluminescent bacteria (data presented in Supplementary Table S9). The results of MDR for the IA modeling are shown in Figure 2, where a clear trend towards overestimation was visible, indicating that sunscreen components compete at the concentrations studied. 

### 2.5. Human Breast Cell Toxicity with MCF10A—A Non-Tumoral, Epithelial Cell Line 

In order to study the possible impact of selected UV filters (EMC and 4MBC) with BPA and DBP (dibutyl phthalate) on human breast cells (with a non-tumoral, epithelial cell line named MCF10A), we first performed dose-response studies on each of the single substances (data not shown), which was followed by experiments using mixtures, performed for 24 and 72 h. The concentrations studied are given in Section 4.6. (for CA and IA models). Both UV filters studied had an antagonistic impact on the BPA and DBP mixture (see Table 1). The IA model was the more reliable one, as the chemicals belonged to different classes. The studies showed a small decrement of magnitude for antagonism with increasing concentrations of DBP in the mixtures with prolonged exposure time, while 4MBC appeared to be a stronger antagonist when compared to EMC.

## 3. Discussion

UV filter compounds are commonly used as active ingredients in many personal care products (PCPs) to protect against sunburns, premature aging and skin cancer caused by the UV light irradiation [17]. In order to ensure PCPs provide adequate protection, a mixture of individual substances, which usually contain three to eight UV filters, is common [3]. Growing concern among consumers about the harmful effects of solar radiation has significantly increased the use of products containing sunscreens, which is directly contributing to their presence in the biome. Currently, there are no regulations inhibiting such combinations [18]. As a consequence, UV filters have been detected in different environmental samples in the ng/L to low μg/L range. For example, OXYB has been detected in seawater samples in the range 13.2 ± 0.4–31.7 ± 0.25 ng/L [19] and <5 to 19.2 μg/L [17]. Moreover, OXYB has been detected in freshwater with quantitative levels ranging from 5 to 79 ng/L, as well as in bottom sediments ranging from <7–82.1 ng/g d.w. [19] and <0.03–65.7 ng/kg d.w. [20]. Some organic UV filters (such as 2-hydroxy-4-methoxybenzophenone, 4-methylbenzylidene camphor and isoamyl 4-methoxycinnamate, among others) have been shown to accumulate in mussels, corals, crabs and fish, with concentrations ranging from few to hundreds of ng/g d.w. (e.g., OXYB and EMC have been detected in cod liver tissue in the range <20–1037 ng/g and <30–36.9 ng/g, respectively [19] (Langford et al., 2015)) and in the livers of dolphins [21].

Our study shows, for the first time, how given selected mixtures of BPs and sunscreen constituents interact in three-component mixtures, at environmentally relevant concentrations. Interestingly, mixtures containing two BPs (BPA + BPS, BPA + BPF, BPS + BPF) and one sunscreen component (OXYB, 4MBC or EMC), show strong synergy in a prevailing number of tests. Moreover, mixtures containing two UV filters (any pair of OXYB, 4MBC and EMC) and one BP (BPA, BPS or BPF) have a strong underestimation potential, which is clearly concentration dependent. The three-component mixtures of UV filters (4MBC, EMC and OXYB) act in an antagonistic manner for each other. Interestingly, the antagonist effects of BPs and these UV filters are conserved in a model human cell line, indicating that these results may be relevant for mammals. We also show that BPF and BPS have as significant impact on the toxicity of these mixtures, which is in agreement with the effects of BPA.

The results show important synergistic interactions between BPs and the three sunscreen constituents, which belong to different chemical classes. The strongest synergy relate to the synergy within three-component mixtures that hold two different types of bisphenols and each of the three sunscreens (see Figure 1A,C,E). The results point out that BPS seems to be important for the synergy action for both OXYB and 4MBC, while the highest doses of EMC are important for the synergy action with the two other BPs. The consistent dose dependency driven by both the BPS and EMC suggest that further studies should be addressed to understand better the mechanisms behind these three-component mixture effects. Overall, our new data suggest that closer attention should be paid to the potential of sunscreens to be much more detrimental in environmentally occurring pollutant mixtures, when compared to their individual effects. The synergy of these UV filters with BPs, which are known to leak out of plastic containers, should prompt investigations into the coexistence of these products in several different kinds of situations. This includes when sunscreens are stored in plastic containers for a long time, sometimes at relatively high temperatures, and then applied to the human skin in relatively high doses [22]. It is relevant, in this context, that sunscreens have the ability to penetrate deep into skin tissues, where they are likely to enable the transfer of other compounds [23,24,25]. For example, in comprehensive skin tests, EMC was shown to penetrate deep into skin tissue. While the penetration was less significant, similar observations have been reported for OXYB and its metabolites [26]. It was also reported that exposure of human macrophages to EMC led to reduced immunity, increasing the risk of asthma and allergy-related complications, due to elevated excretion of cytokines [27]. Limited studies can be found on the interactions between sunscreen constituents and other pollutants [28]. In the work of Brand et al. [29], scientists confirmed enhanced penetration of selected pesticides (e.g., 2,4-D (2,4-Dichlorophenoxyacetic acid), DEET (N,N-diethyl-m-toluamide), paraquat, parathion and malathion) when hairless mouse skin was co-exposed to titanium and zinc oxides [29]. In the work of Marrot [30], the possible explanation for elevated atopy or eczema during periods of increased pollution exposure (heavy metals or polycyclic aromatic hydrocarbons (PAHs)) include oxidative stress, inflammation and metabolic impairments correlated to more frequent use of sunscreens. It would be important to investigate if BPs can potentially contribute to such sunscreen-related effects.

The antagonistic actions of these pollutants at environmentally relevant doses are also potentially very important. Our finding that three-component mixtures of UV filters (4MBC, EMC and OXYB) act in an antagonistic manner highlights their potential underestimation for biological consequences. Moreover, we found that such antagonist actions could be replicated in a human breast cell model, suggesting this may also be relevant for mammalian models, something that is worth further experimental consideration. Such antagonist effects can potentially skew conclusions of testing in different biological models. We also highlight the importance of using different experimental models to confirm such findings. Our study highlights the importance of dose dependency for both these types of pollutants, justifying the necessity to perform mixture studies using a wide range of concentrations. 

Mixtures of UV filters and BPs have never been studied before with the help of marine organisms, and these results open up a new field for experimental design. Until now, only single pollutants belonging to UV filters have been studied with microorganisms belonging to different trophic levels. OXYB appears to be the most toxic substance among the UV filters, considering studies on microalgae (*I. galbana*), where OXYB was found to have an EC50 of 13.87 µg/L, followed by EMC (EC50 74.73 µg/L) and 4MBC (EC50 171.5 µg/L) [31]. In another model, employing Mediterranean mussel (*M. galloprovincialis*), the 4MBC solution (EC50 587 µg/L) was found to be the most toxic among the UV filters studied, while slightly lower toxicities were found for EMC (EC50 3110 µg/L) and OXYB (EC50 3470 µg/L). Studies using sea urchins (*P. lividus*) showed that this organism was more susceptible to EMC (EC50 284 µg/L) and 4MBC (EC50 854 µg/L), when compared with exposure to OXYB (EC50 3280 µg/L) [32]. Based on these results, it can be suggested that each marine organism responds to BPs and sunscreens components in a different manner. Individual predispositions and environmental conditions may also contribute, but further studies investigating the co-present pollutants are warranted to understand real-world consequences.

4MBC is known to cause detrimental effects in several test models. For example, 4MBC has been extensively investigated in terms of impact on reproductive systems. The Japanese rice fish, also known as the medaka (*Oryzias latipes*), is a fresh and brackish water fish, making it a good model for upper-tier ecotoxicological battery testing. Exposing adult male medaka to 4MBC resulted in disruptive spermatogenesis at doses of 5 and 50 μg/L. While 5 μg/L solutions greatly impacted estradiol and vitellogenin concentrations in the female plasma [33]. Furthermore, exposure of medaka eggs to a 4MBC solution resulted in prolonged hatching times. Japanese mussels (*Ruditapes philippinarum*) exposed to 4MBC at concentrations from 1–100 µg/L resulted in physiological stress due to elevated levels of antioxidant enzymes (GST) and proteins related to the inhibition of apoptosis (BCL2) and cellular stress (GADD). Sea snail larvae (*Sinum vittatum*) exposed to 2.57 mg/kg solutions of 4MBC had a decreased harvesting rate, while oxygen stress indicators were not impaired in these test organisms [34]. Moreover, the cell viability, oxidative stress and growth impairments as toxicological endpoints were studied with *Tetrahymena thermophile* protozoans [35] exposed separately to 4MBC and EMC. The EC_50_ of 4MBC, after 24 h exposure, reached 5.125 mg/L, while toxicological endpoints of growth inhibition and cells vitality (5 mg/L at 6 h) were correlated with an ability to break down cellular membranes at 15 mg/L after 4 h post exposure. 4MBC has also been studied in mammals, including humans and rats, where plasma concentration levels were measured after application of a sunscreen product (containing 4% of 4MBC) to the skin [36]; the maximum plasma values reached 200 pmol/mL. Only a small portion of 4MBC glucuronide metabolite was detected in human urea samples, confirming the low metabolic capability of humans in 4MBC transformation processes. Bearing in mind all of the above, it becomes evident how important it is to study CEC mixtures in various organisms and cell lines, which belong to different trophic levels, as pollutant cocktails greatly lower effective concentration values in the case of synergy confirmation. 

The UV filters and BPs we tested are commonly found together in numerous water bodies [37]. Large studies, performed to determine over 100 CECs, confirmed the co-presence of sunscreen active components and BPs in effluents from wastewater treatment plants [38], although in lower concentrations than studied here. Oxybenzone and BPs were also instrumentally determined in samples collected from the Niger River delta [39], confirming a necessity for considering any environmental sample as a cocktail of numerous unknown ingredients and their metabolites/transformation products. Certainly, more work is needed to learn the mechanism of the enhanced toxicity of mixtures studied here, but our findings suggest that the action of a mixture of UV filters and BPs interfere with each other. Mixtures containing any of the two BPs studied and one sunscreen show a clear tendency towards underestimation and synergy, while the effect for cocktails containing any two UV filters studied and one BP show a slightly weaker effect. Overall, we find that underestimation events seem to be more frequent and there is a clear concentration dependence trend. Our results suggest that more studies looking at the direct interaction of these sunscreen and BP molecules with their potential cellular protein targets are warranted. 

## 4. Materials and Methods

### 4.1. Chemicals and Reagents

Three different organic UV-filters were selected in this study, i.e., OXYB (oxybenzone, CAS no. 131-57-7), 4MBC (4-methylbenzylidene-camphor, CAS no. 36861-47-9) and EMC (2-ethylhexyl 4-methoxycinnamate, CAS no. 5466-77-3), and three bisphenol-analogue compounds, i.e., BPA (bisphenol A, CAS no. 80-05-7), BPF (bisphenol F, CAS no. 620-92-8) and BPS (bisphenol S, CAS no. 80-09-1), which were purchased from Sigma Aldrich (Darmstadt, Germany). HPLC grade MeOH (methanol, CAS no. 67-56-1) was purchased from Merc (KGaA, Darmstadt, Germany). Ultrapure water was produced by a Milli-Q Gradient A10 system equipped with an EDS-Pak cartridge to remove endocrine disrupting compounds (Merck–Millipore, Darmstadt, Germany). 

Chemicals used for Microtox^®^ were purchased from Modern Water Ltd. (New Castle, DE, USA). These were 2% NaCl solution, lyophilized *Aliivibrio fischeri* bacteria, Microtox Diluent, Osmotic Adjusting Solution (OAS) and Reconstitution Solution (RS). The instruments and equipment used during the studies were Microtox^®^ 500 analyser of Modern Water Ltd. (Modern Water Ltd., USA) and electronic pipettes (Mettler Toledo, Columbus, OH, USA; Eppendorf, Hamburg, Germany).

The breast cell line MCF10A was donated by Professor Anna-Karin Olsson from the Dept. of Medical Biochemistry and Microbiology (IMBIM), Uppsala Biomedical Center (BMC), Sweden. MCF10A cells were maintained as a monolayer in T-75 cell culture plastic flasks (Corning Life Science, Amsterdam, the Netherlands) containing 12 mL of growth medium, trypsinized (Trypsin, no phenol red, Gibco™, Catalog number: 15090046) and split 1:4 every 3 days. Complete growth medium consisted of Dulbecco’s Modified Eagle Medium with F-12 (DMEM/F-12, Gibco™, Catalog number: 31331028) supplemented with 5% horse serum (Horse Serum, heat inactivated, Gibco™, Catalog number: 26050088), 0.5 mg/mL hydrocortisone (Sigma-Aldrich, H0888, (Stockholm, Sweden)), 100 ng/mL CT (Cholera Toxin, Sigma-Aldrich, C8052), 10 mg/mL insulin (Sigma-Aldrich, I6634), EGF (20 ng/mL, Human Epidermal Growth Factor (EGF) Recombinant Protein, Gibco™, Catalog number: PHG0311) and 5 mL P/S (Penicillin-Streptomycin, Gibco™, Catalog number: 15140148 ()). Cell cultures were maintained at 37 °C and 5% CO_2_ in a humidified incubator.

### 4.2. Preparation of Model Solutions

Before starting the tests, working solutions (C1 = 5 µM, C2 = 10 µM, C3 = 20 µM) were prepared by diluting the stock solutions with MeOH and ultrapure Milli-Q water, previously made by dissolving accurately weighted amounts of analytical standards in HPLC grade MeOH (4 mg/mL). All individual stock solutions were stored in the dark at −20 °C. The working solutions were freshly prepared before each set of analysis. The amount of MeOH was set at a maximum of 2% in these solutions for all toxicity tests and was used as the solvent control medium. In Table 2, the basic information on the studied sunscreens and BPA analogues are given [21,40].

### 4.3. Preparation of Ternary Mixtures

The mixtures were prepared via mixing three different compounds diluted to requested concentrations. The general scheme used to prepare the mixtures is shown below. The same approach followed suitably for C2 and C3 of S1 (C stands for concentration and S for substance/analyte in mixture). In this way, all mixtures were prepared and studied in duplicated experiments as follows: 

Mixture 1: S1C1 + S2C1 + S3C1

Mixture 2: S1C1 + S2C1 + S3C2

Mixture 3: S1C1 + S2C1 + S3C3

Mixture 4: S1C1 + S2C2 + S3C1

Mixture 5: S1C1 + S2C2 + S3C2

Mixture 6: S1C1 + S2C2 + S3C3

Mixture 7: S1C1 + S2C3 + S3C1

Mixture 8: S1C1 + S2C3 + S3C2

Mixture 9: S1C1 + S2C3 + S3C3

Mixture 10: S1C2 + S2C1 + S3C1

Mixture 11: S1C2 + S2C1 + S3C2

Mixture 12: S1C2 + S2C1 + S3C3

Mixture 13: S1C2 + S2C2 + S3C1

Mixture 14: S1C2 + S2C2 + S3C2

Mixture 15: S1C2 + S2C2 + S3C3

Mixture 16: S1C2 + S2C3 + S3C1

Mixture 17: S1C2 + S2C3 + S3C2

Mixture 18: S1C2 + S2C3 + S3C3

Mixture 19: S1C3 + S2C1 + S3C1

Mixture 20: S1C3 + S2C1 + S3C2

Mixture 21: S1C3 + S2C1 + S3C3

Mixture 22: S1C3 + S2C2 + S3C1

Mixture 23: S1C3 + S2C2 + S3C2

Mixture 24: S1C3 + S2C2 + S3C3

Mixture 25: S1C3 + S2C3 + S3C1

Mixture 26: S1C3 + S2C3 + S3C2

Mixture 27: S1C3 + S2C3 + S3C3 

In parallel with mixtures, studies using single compounds in respective, environmentally relevant concentrations were performed for every experiment run (experiments were performed on a daily routine for the same standards, solutions and bacterial reagents batch). The raw dose-response data for the studied compounds are presented in electronic Supplementary Materials as Figure S1.

### 4.4. Acute Toxicity Determination of the Mixtures

In order to determine the toxicity of the analytes of interest and their ternary mixtures, a standard Microtox^®^ acute assay has been performed. To achieve a ready-to-use bacterial suspension, the lyophilized *Aliivibrio fischeri* bacteria were rehydrated with 1 mL of RS. The cell suspension was immediately transferred into a glass cuvette placed in the reagent well of the analyzer maintained at 5.5 ± 1.0 °C. Subsequently, 100 µL of bacterial solution and 900 µL of working solutions were added into the test vials. Before starting the test, an osmotic adjustment was performed, using a saline solution to make the sample salinity optimal (above 2%) for *Aliivibrio fischeri*. The cuvettes were incubated at 15 °C. The toxicity was determined based on the inhibition of the light naturally emitted (at 490 nm wavelength) by the bacteria after its exposure to a standard solution/mixture sample. The bioluminescence level was detected by Microtox^®^ Model 500 Analyzer. Measurements of the luminescent output of the bacteria were recorded and compared with the light output of the control sample after 30 min. Each assay was performed in a duplicate. For quality control—according to delivery certificates—the phenol and copper (II) sulphate were used as positive controls.

Change of bioluminescence after *t* time was calculated according to Equation (1):

% bioluminescence change *_t_* = [G*_t_*/(1+ G*_t_*)] · 100 (1)

where G (gamma correction factor) was calculated with Equation (2):

G*_t_* = [(R_*t*_ · I_0_)/I_*t*_] − 1 (2)

where I_*t*_ is the bioluminescence of bacteria in real sample at time of *t*, I_0_ is the initial bioluminescence of bacteria in real samples and R_*t*_ is calculated with Equation (3):

R_*t*_ = Ic_*t*_/Ic_0_ (3)

where I_c*t*_ is the bioluminescence of bacteria in control sample after *t* time of incubation and I_c0_ is the initial bioluminescence of bacteria in control.

### 4.5. Modeling and MDRs Calculations

The modeling calculations and MDR have been done according to standardized procedures described in detail in [41]. Mathematical modeling has been performed with concentration addition (CA) and independent action (IA) approaches, followed by the data interpretation with the Model Deviation Ratio (MDR) evaluation as presented in details in [42]. Here, the CA was assessed by using Equation (4):

(4) $$ECx_{mix}={\left(\sum_{i=0}^{n}\frac{p_{i}}{ECx_{i}}\right)}^{-1}$$

where *ECx_mix_* is the total concentration of the mixture that causes *x* effect, *p_i_* indicates the proportion of component *i* in the mixture, *n* indicates the number of components in the mixture and *ECx_i_* indicates the concentration of component *i* that would cause *x* effect.

The independent action model is used to test toxicants in a mixture for a dissimilar mode of action, assuming that they act independently; the IA model is a statistical approach to predict the chance that one of multiple events will occur. The total mixture effect is calculated using Equation (5):

(5) $$E\left(c_{mix}\right)=1-\prod_{i=0}^{n}\left(1-E\left(c_{i}\right)\right)$$

where *E*(*c_mix_*) is the total concentration of the mixture and *E*(*c_i_*) is the concentration expected from component *i*.

The CA model does not account for a possible interaction between different chemicals in the mixture and the deviations of tested mixture toxicity from the predicted one could be evidence for synergistic or antagonistic interaction between chemicals. To outline significant deviations (interactions between chemicals) the model deviation ratio (MDR) approach is applied. MDR (unitless) is defined with Equation (6):

(6) $$MDR=\frac{Expected\;toxicity}{Observed\;toxicity}$$

where *Expected toxicity* is the effective concentration toxicity for the mixture predicted by the CA/IA model and *Observed toxicity* is the effective concentration toxicity for the mixture obtained from toxicity testing.

MDR values below 0.5 indicate the presence of synergism, values in the range 0.5–0.71 indicate underestimation of the model, values in the range 1.4–2.0 indicate overestimation of the model and values above 2.0 indicate antagonism. All calculations were performed with Microsoft Excel 2016 standard set. 

### 4.6. Methodology of Human Breast Cell Line Studies

Human breast epithelial cells (MCF10A) were seeded in 250 µL complete growth medium within the inner wells of a 96-well plate (Corning Life Science, Amsterdam, the Netherlands) at a density of approximately 2 × 10^4^ cells/well. The outer wells were excluded from the experiment and filled in with PBS. To permit cell adhesion and adaption to the novel environment, the cells were placed in a humidified incubator at 37 °C and 5% CO_2_ for 24 h. After 24 h, the cells were exposed to different combination of pollutant mixtures (C1 = 1 µM, C2 = 5 µM and C3 = 10 µM), which were dissolved in DMSO (dimethyl sulfoxide, Invitrogen™, Catalog number: D12345). Then, after 24 and 72 h (in different trials), PrestoBlue™ HS Cell Viability Reagent (Invitrogen™, Catalog number: P50200. Pub. No. MAN0018371) was employed to test the cell viability, which reflects cell proliferation. The data were further analyzed in Microsoft Excel and GraphPad Prism (version 9.0.0). Each treatment was conducted with at least three biological replicates.

## 5. Conclusions

The increasing emissions of CECs into the environment prompts scientists to intensify their research related to the determination of interactions between chemicals co-present in complex matrices. Mixtures of CECs are found in practically all industrial and public service branches; from pharmaceutics, health, wastewater treatment, catalyst applications, ecosystem monitoring, life cycle assessment and many others. Thus, it is increasingly important not only to develop new tools to instrumentally determine the content of given CEC mixtures, but also to validate and adjust already known approaches. Further development of advanced mathematical tools to confirm possible interactions that occur between pollutants are warranted, especially for mixtures of higher orders. 

This study is one of the most comprehensive on the interactions of compounds in complex mixtures, providing evidence for the notion that concentrations of a given CEC plays a crucial, and sometimes unpredictable, role in exerting cellular or physiological impacts on a living organism. Our mathematical approach and experimental setup enable the collection of rational and reliable data, which prompt conclusions on how to assess the potential overadditive or canceling effects of cocktail components. The findings presented here are an important next step for a better understanding of how to perform complex toxicological studies in a systematic manner, and how to evaluate a model’s validity relating to dissimilar groups of pollutants and cells/organisms belonging to different trophic levels.

In our opinion, this work adds new insights into the field of mixtures impact on biota and confirms the necessity to increase the number of studies on complex mixtures of chemicals affecting biota and, gradually, start introducing requirements on admissible concentrations of particular chemicals, bearing in mind their toxicological impact on biota when present in mixtures. In such cases, law and policy makers need reliable sources of information to present and suggest realistic and achievable goals in directives aimed at minimizing the impact of versatile pollutant cocktails on humans.

## Figures and Tables

**Table 1:** MDR results of CA and IA toxicity modeling with MCF10A human breast cancer cells (MDR values > 2.0 exhibit antagonism, MDRs < 0.5 show synergism and MDR values of 0.50–0.71 and 1.40–2.00 mean, respectively, under- and overestimation of the presented models *, *n* = 3).

	S3: EMC				S3: 4MBC			
	24 h		72 h		24 h		72 h	
	CA	IA	CA	IA	CA	IA	CA	IA
C1 BPA + C1 DBP + S3 C1	0.923	2.728	0.852	2.521	0.980	2.897	1.003	2.964
C1 BPA + C1 DBP + S3 C3	0.921	2.708	0.836	2.553	0.970	2.879	1.033	3.018
C1 BPA + C2 DBP + S3 C1	0.869	2.543	0.835	2.486	0.931	2.722	0.856	2.573
C1 BPA + C2 DBP + S3 C3	0.851	2.500	0.774	2.355	0.932	2.756	0.869	2.576
C1 BPA + C3 DBP + S3 C1	0.857	2.492	0.780	2.327	0.907	2.632	0.829	2.506
C1 BPA + C3 DBP + S3 C3	0.840	2.464	0.761	2.309	0.895	2.634	0.856	2.563

* red color indicates confirmed synergy, blue indicates antagonism, while yellow and green refer to under- and overestimation, respectively.

**Table 2:** Basic information on the UV filters and BPs studied.

Compound/Analyte	Acronym	CAS no.	Formula	Chemical Structure	Molecular Weight	logP	pKa	logK_ow_	Water Solubility [mg/L]
Sunscreen components:									
Oxybenzone	OXYB	131-57-7	C_14_H_12_O_3_	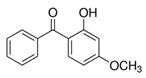	228.24	3.79	7.56	3.79	3.7 (at 25 °C)
4-Methylbenzylidene-camphor	4MBC	36861-47-9	C_18_H_22_O	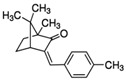	254.37	5.14	>8	4.95	1.3 (at 20 °C)
2-Ethylhexyl 4-methoxycinnamate	EMC	5466-77-3	C_18_H_26_O_3_	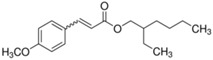	290.40	5.66	-	5.80	<0.15 (at 25 °C)
Bisphenols:									
2,2-Bis(4-hydroxyphenyl)propane	BPA	80-05-7	C_15_H_16_O_2_	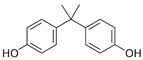	228.29	4.04	9.78–10.39	3.43	120 (at 20 °C)
4,4′-Methylenediphenol	BPF	620-92-8	C_13_H_12_O_2_	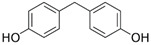	200.23	2.9	9.84–10.45	2.76	200 (at 25 °C)
4,4′-Sulfonyldiphenol	BPS	80-09-1	C_12_H_10_O_4_S	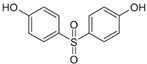	250.27	2.32	7.42–8.03	2.139	350 (at 25 °C)

## Data Availability

Data can be shared upon personal request to the authors.

## Supplementary Materials

The following supporting information can be downloaded at: https://www.mdpi.com/article/10.3390/molecules27103260/s1, Figure S1. The raw dose-response data for (a) BPA, (b) BPS, (c) BPF, (d) OXYB, (e) EMC and (f) 4-MBC that were used to pre-select concentration levels of mixtures components in this research (bars represent SD values for *n* = 2); Figure S2. MDR values of bioluminescent bacteria results for: (A) CA modeling of the BPS, OXYB and BPF mixture, (B) CA modeling of the BPS, OXYB and 4MBC mixture, (C) CA modeling of the BPS, OXYB and EMC mixture (D) CA modeling of the BPS, 4MBC and EMC mixture, (E) CA modeling of the BPS, EMC and BPF mixture (*n* = 2). Red color indicates confirmed synergy, blue indicates antagonism, while yellow and green refer to under- and overestimation, respectively; Figure S3. MDR values of bioluminescent bacteria results for: (A) CA modeling of the BPF, 4MBC and BPS mixture, (B) CA modeling of the BPF, 4MBC and EMC mixture, (C) CA modeling of the BPF, 4MBC and OXYB mixture (D) CA modeling of the BPF, OXYB and EMC mixture (*n* = 2). Red color indicates confirmed synergy, blue indicates antagonism, while yellow and green refer to under- and overestimation, respectively; Supplementary Table S1. MDR results of studies on the impact of three-component mixtures on BPA, BPS, BPF, 4MBC, EMC and OXYB toxicity (MDR values >2.0 exhibit antagonism, MDRs < 0.5 show synergism and MDR values of 0.50–0.71 and 1.40–2.00 mean, respectively, under- and overestimation of the presented models; for values of particular concentrations C1, C2 and C3 of all analytes, please refer to Section 2.2. in the main text) (*n* = 2); Supplementary Table S2. MDR results of studies on the impact of three-component mixtures on BPA, BPS, BPF, 4MBC and EMC toxicity (MDR values > 2.0 exhibit antagonism, MDRs < 0.5 show synergism and MDR values of 0.50–0.71 and 1.40–2.00 mean, respectively, under- and overestimation of the presented models; for values of particular concentrations C1, C2 and C3 of all analytes, please refer to Section 2.2. in the main text) (*n* = 2); Supplementary Table S3. MDR results of studies on the impact of three-component mixtures on BPA, BPS, BPF, 4MBC and EMC toxicity (MDR values > 2.0 exhibit antagonism, MDRs < 0.5 show synergism and MDR values of 0.50–0.71 and 1.40–2.00 mean, respectively, under- and overestimation of the presented models; for values of particular concentrations C1, C2 and C3 of all analytes, please refer to Section 4.2. in the main text) (*n* = 2); Supplementary Table S4. MDR results of studies on the impact of three-component mixtures on BPA, BPS, BPF, 4MBC and EMC toxicity (MDR values > 2.0 exhibit antagonism, MDRs < 0.5 show synergism and MDR values of 0.50–0.71 and 1.40–2.00 mean, respectively, under- and overestimation of the presented models; for values of particular concentrations C1, C2 and C3 of all analytes, please refer to Section 4.2. in the main text) (*n* = 2); Supplementary Table S5. MDR results of studies on the impact of three-component mixtures on BPA, BPS, BPF, 4MBC and EMC toxicity (MDR values > 2.0 exhibit antagonism, MDRs < 0.5 show synergism and MDR values of 0.50–0.71 and 1.40–2.00 mean, respectively, under- and overestimation of the presented models; for values of particular concentrations C1, C2 and C3 of all analytes, please refer to Section 4.2. in the main text) (*n* = 2); Supplementary Table S6. MDR results of studies on the impact of three-component mixtures on BPS, BPF and EMC toxicity (MDR values > 2.0 exhibit antagonism, MDRs < 0.5 show synergism and MDR values of 0.50–0.71 and 1.40–2.00 mean, respectively, under- and overestimation of the presented models; for values of particular concentrations C1, C2 and C3 of all analytes, please refer to Section 4.2. in the main text) (*n* = 2); Supplementary Table S7. MDR results of studies on the impact of three-component mixtures on BPF, 4MBC, BPS, OXB and EMC toxicity (MDR values > 2.0 exhibit antagonism, MDRs < 0.5 show synergism and MDR values of 0.50–0.71 and 1.40–2.00 mean, respectively, under- and overestimation of the presented models; for values of particular concentrations C1, C2 and C3 of all analytes, please refer to Section 4.2. in the main text) (*n* = 2); Supplementary Table S8. MDR results of studies on the impact of three-component mixtures on BPA, BPS, BPF, 4MBC and EMC toxicity (MDR values > 2.0 exhibit antagonism, MDRs < 0.5 show synergism and MDR values of 0.50–0.71 and 1.40–2.00 mean, respectively, under- and overestimation of the presented models; for values of particular concentrations C1, C2 and C3 of all analytes, please refer to Section 4.2. in the main text) (*n* = 2); Supplementary Table S9. MDR results of studies on the impact of three-component mixtures on OXYB, 4MBC and EMC toxicity (MDR values > 2.0 exhibit antagonism, MDRs < 0.5 show synergism and MDR values of 0.50–0.71 and 1.40–2.00 mean, respectively, under- and overestimation of the presented models; for values of particular concentrations C1, C2 and C3 of all analytes, please refer to Section 4.2. in the main text) (*n* = 2).

## Sample Availability

Samples of the compounds studied are available from the commercial producers.
