# Supplementary material for: Enhanced Toxicity of Bisphenols Together with UV Filters in Water: Identification of Synergy and Antagonism in Three-Component Mixtures
Source: Molecules. 2022 May 19;27(10):3260. doi: 10.3390/molecules27103260 (PMC9143986; doi:10.3390/molecules27103260)
Supplement: Supplementary file 1 [file molecules-27-03260-s001.zip › molecules-1558204-supplementary.pdf]

Enhanced toxicity of bisphenols together with UV filters in water. Identification of synergy and antagonism in three component mixtures

Błażej Kudłak[1], Natalia Jatkowska[1*], Wen Liu[2], Michael J. Williams[2], Damia Barcelo[3], Helgi B. Shioth[2]

[1]Department of Analytical Chemistry, Faculty of Chemistry, Gdańsk University of Technology, 11/12 Narutowicza Str., Gdańsk 80-233, Poland

[2] Functional Pharmacology, Department of Neuroscience, Uppsala University, 751 24 Uppsala, Sweden

[3] ICRA, Catalan Institute for Water Research (ICRA), Parc Científic i Tecnològic de la Universitat de Girona, C/ Emili Grahit, 101 Edifici H2O, E-17003 Girona, Spain

* - Corresponding Authors: natjatko@pg.edu.pl

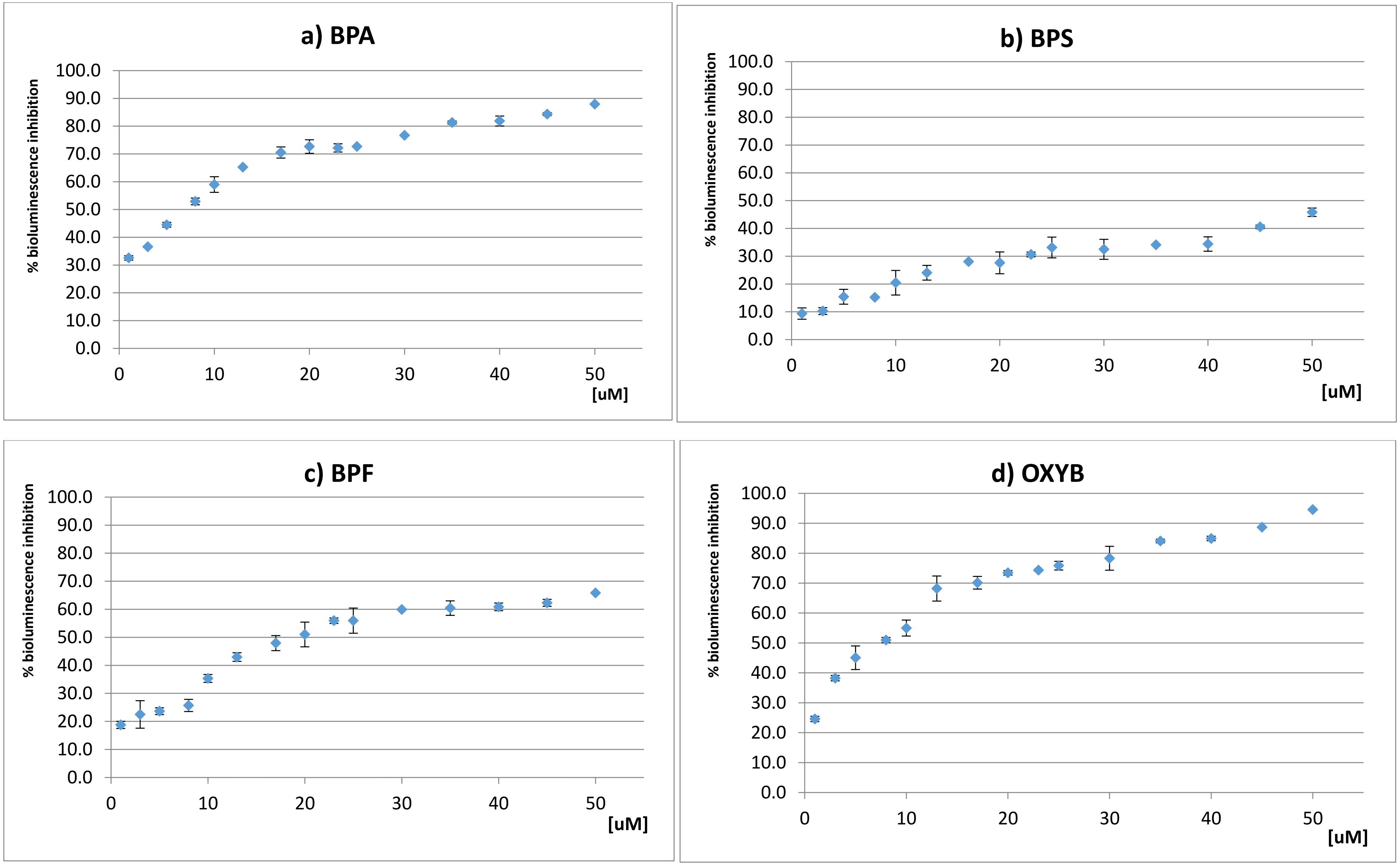
a) BPA
b) BPS
c) BPF
d) OXYB
% bioluminescence inhibition
[uM]
0.0
10.0
20.0
30.0
40.0
50.0
60.0
70.0
80.0
90.0
100.0
0
10
20
30
40
50

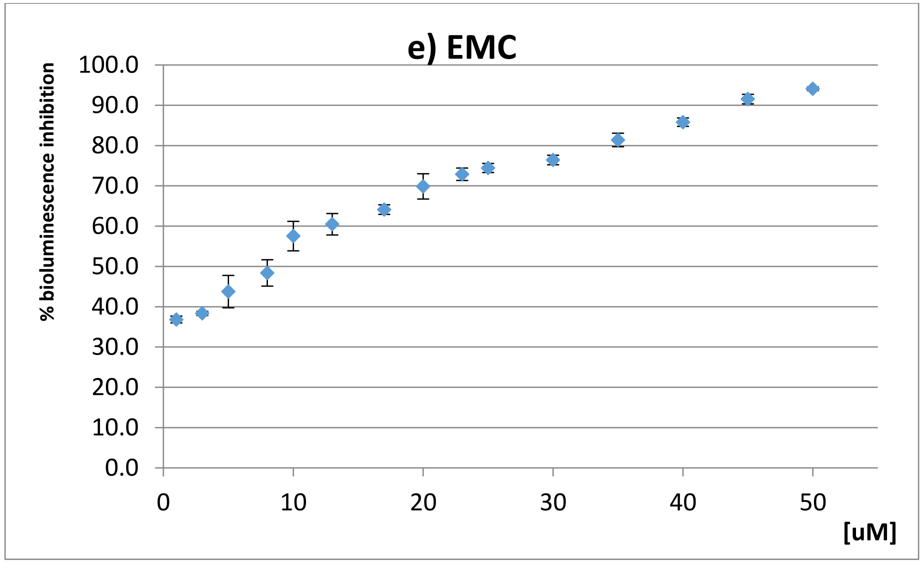

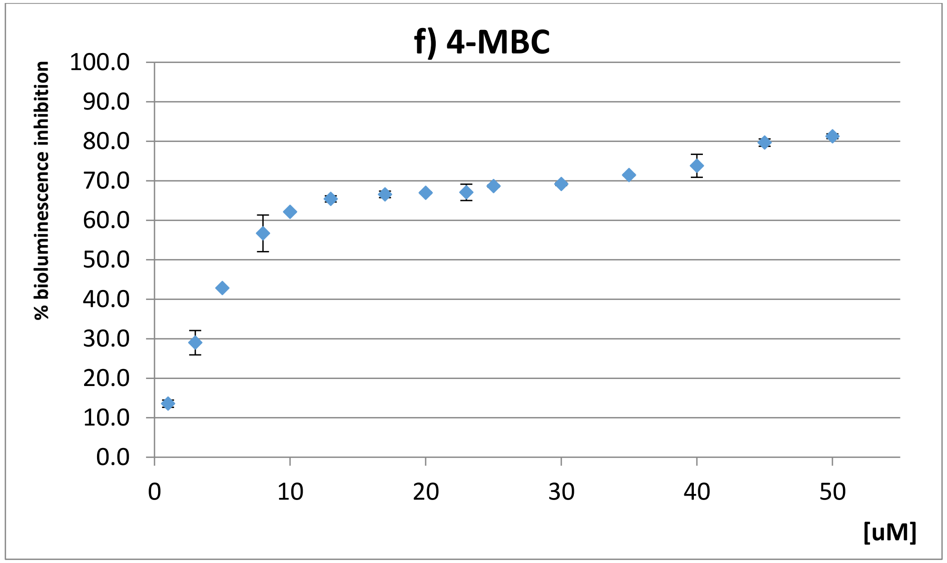

Figure S1. The raw dose-response data for the a) BPA, b) BPS, c) BPF, d) OXYB, e) EMC, f) 4-MBC that were used to pre-select concentration levels of mixtures components in this research (bars represent SD values for n=2).

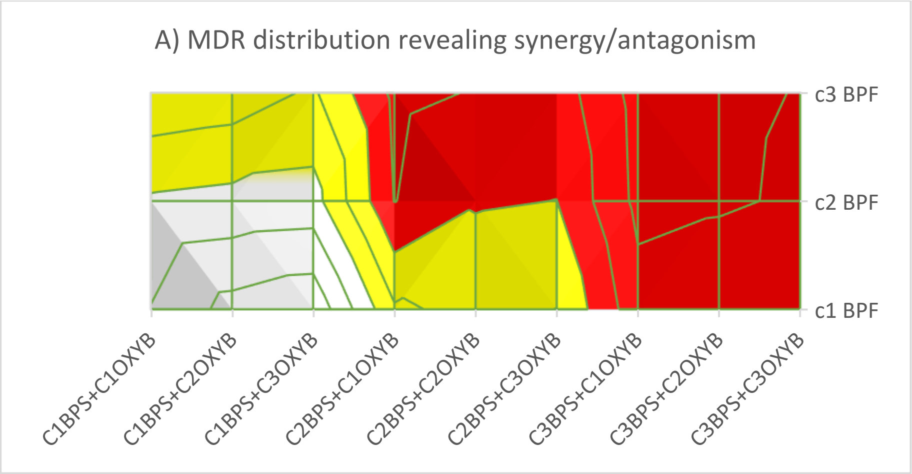
A) MDR distribution revealing synergy/antagonism
c3 BPF
c2 BPF
c1 BPF
C1BPS+C1OXYB
C1BPS+C2OXYB
C1BPS+C3OXYB
C2BPS+C1OXYB
C2BPS+C2OXYB
C2BPS+C3OXYB
C3BPS+C1OXYB
C3BPS+C2OXYB
C3BPS+C3OXYB

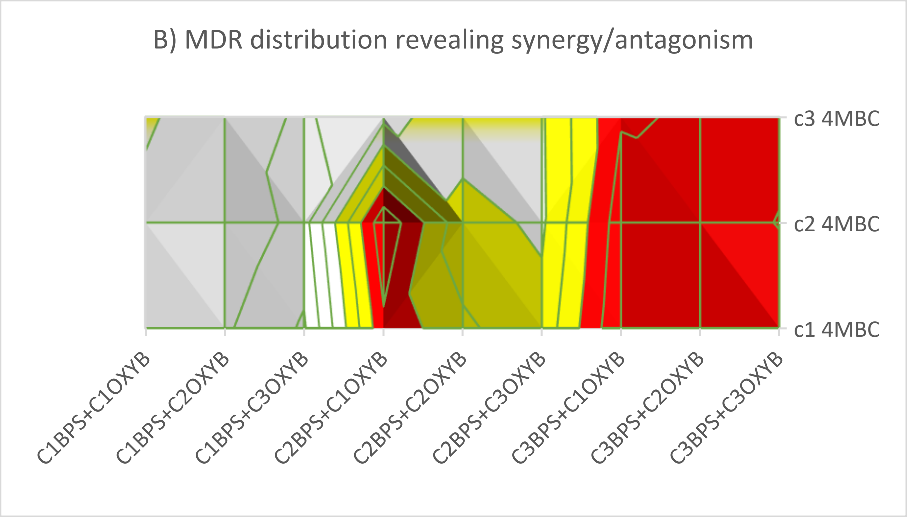
B) MDR distribution revealing synergy/antagonism
c3 4MBC
c2 4MBC
c1 4MBC
C1BPS+C1OXYB
C1BPS+C2OXYB
C1BPS+C3OXYB
C2BPS+C1OXYB
C2BPS+C2OXYB
C2BPS+C3OXYB
C3BPS+C1OXYB
C3BPS+C2OXYB
C3BPS+C3OXYB

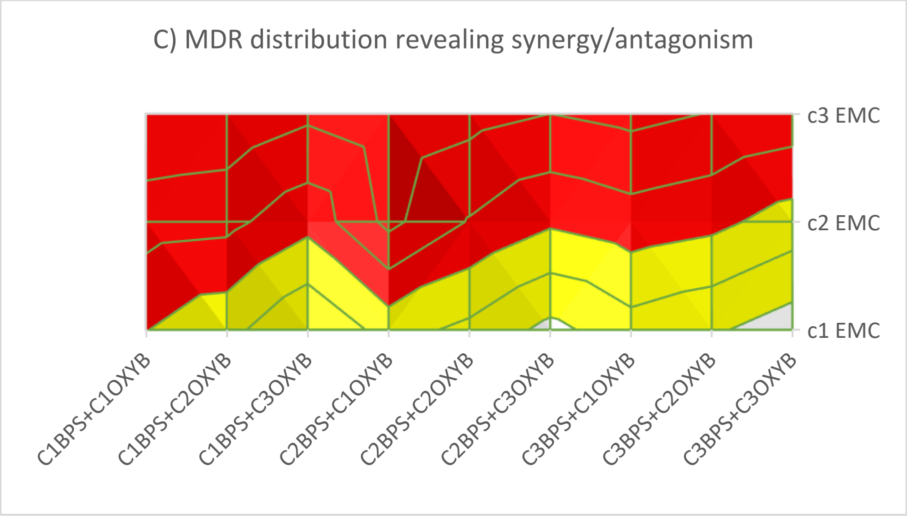
C) MDR distribution revealing synergy/antagonism
c3 EMC
c2 EMC
c1 EMC
C1BPS+C1OXYB
C1BPS+C2OXYB
C1BPS+C3OXYB
C2BPS+C1OXYB
C2BPS+C2OXYB
C2BPS+C3OXYB
C3BPS+C1OXYB
C3BPS+C2OXYB
C3BPS+C3OXYB

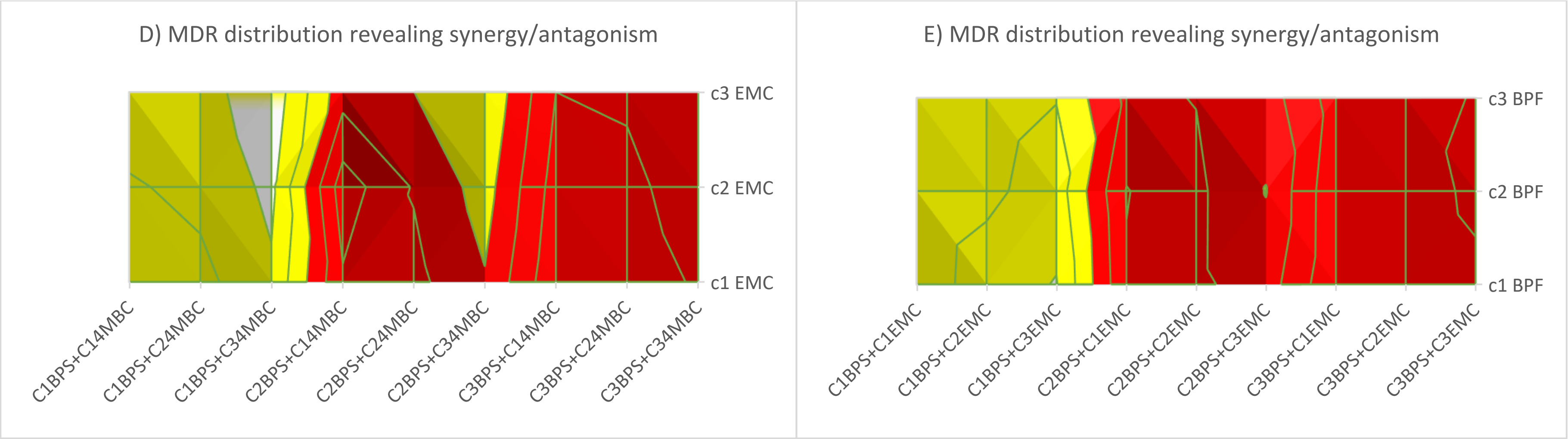

Figure S2. MDR values of bioluminescent bacteria results for: A) CA modelling of BPS, OXYB and BPF mixture, B) CA modelling of BPS, OXYB and 4MBC mixture, C) CA modelling of BPS, OXYB and EMC mixture D) CA modelling of BPS, 4MBC and EMC mixture, E) CA modelling of BPS, EMC and BPF mixture (n=2). Red color indicates confirmed synergy, blue – antagonism while yellow and green refer to under- and overestimation, respectively.

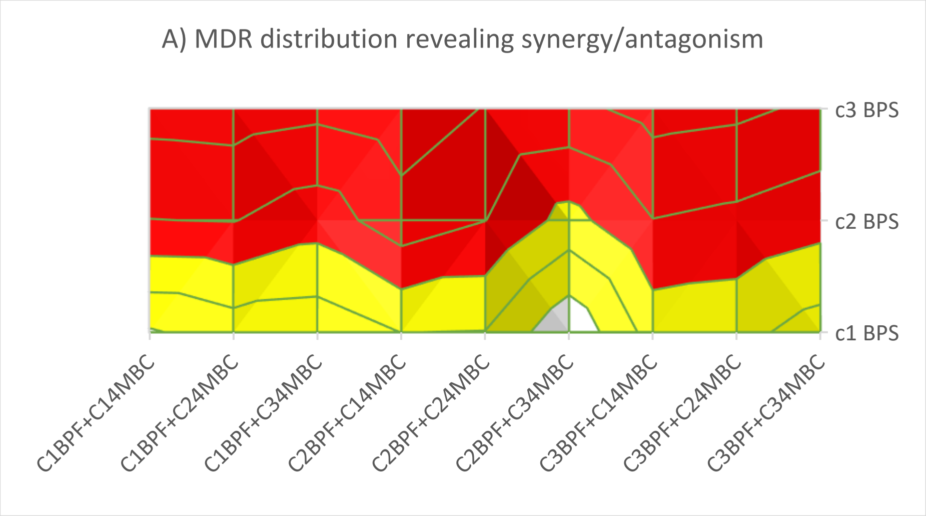
A) MDR distribution revealing synergy/antagonism
c3 BPS
c2 BPS
c1 BPS
C1BPF+C14MBC
C1BPF+C24MBC
C1BPF+C34MBC
C2BPF+C14MBC
C2BPF+C24MBC
C2BPF+C34MBC
C3BPF+C14MBC
C3BPF+C24MBC
C3BPF+C34MBC

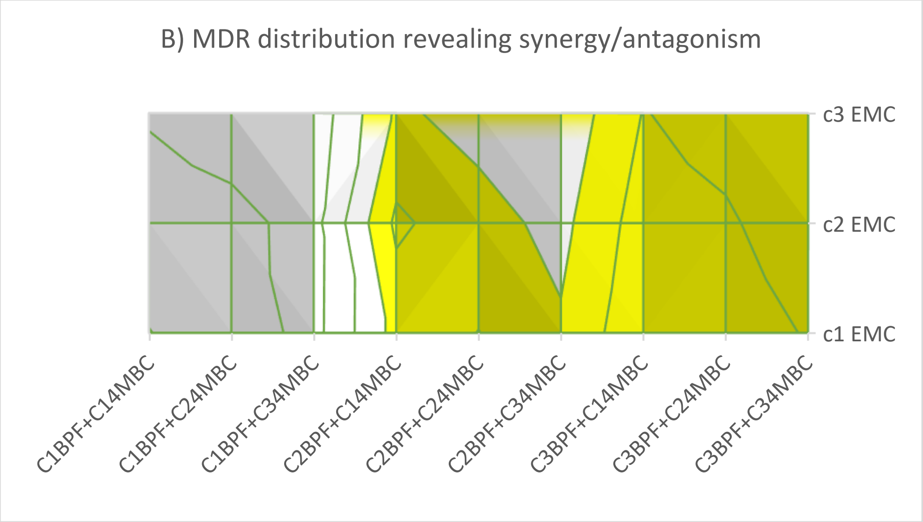
B) MDR distribution revealing synergy/antagonism
c3 EMC
c2 EMC
c1 EMC
C1BPF+C14MBC
C1BPF+C24MBC
C1BPF+C34MBC
C2BPF+C14MBC
C2BPF+C24MBC
C2BPF+C34MBC
C3BPF+C14MBC
C3BPF+C24MBC
C3BPF+C34MBC

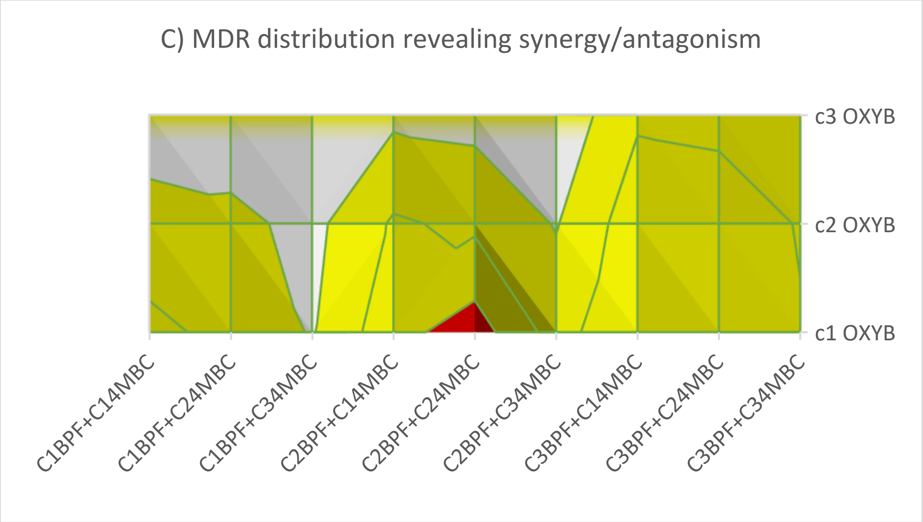
C) MDR distribution revealing synergy/antagonism
c3 OXYB
c2 OXYB
c1 OXYB
C1BPF+C14MBC
C1BPF+C24MBC
C1BPF+C34MBC
C2BPF+C14MBC
C2BPF+C24MBC
C2BPF+C34MBC
C3BPF+C14MBC
C3BPF+C24MBC
C3BPF+C34MBC

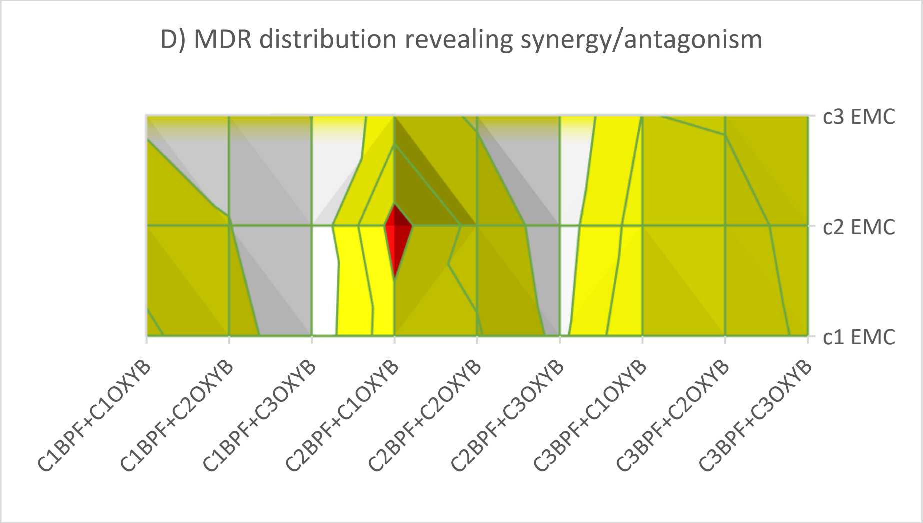
D) MDR distribution revealing synergy/antagonism
c3 EMC
c2 EMC
c1 EMC
C1BPF+C1OXYB
C1BPF+C2OXYB
C1BPF+C3OXYB
C2BPF+C1OXYB
C2BPF+C2OXYB
C2BPF+C3OXYB
C3BPF+C1OXYB
C3BPF+C2OXYB
C3BPF+C3OXYB

Figure S3. MDR values of bioluminescent bacteria results for: A) CA modelling of BPF, 4MBC and BPS mixture, B) CA modelling of BPF, 4MBC and EMC mixture, C) CA modelling of BPF, 4MBC and OXYB mixture D) CA modelling of BPF, OXYB and EMC mixture (n=2). Red color indicates confirmed synergy, blue – antagonism while yellow and green refer to under- and overestimation, respectively.

Supplementary Table S1. MDR results of studies on impact of three component mixture on BPA, BPS, BPF, 4MBC, EMC and OXYB toxicity (MDR values >2.0 exhibit antagonism, MDRs <0.5 show synergism, MDR values within 0.50–0.71 and 1.40–2.00 values mean, respectively, under- and overestimation of presented models; for values of particular concentrations C1, C2 and C3 of all analytes, please refer to subchapter 4.2. in the main text) (n=2)

		BPS						BPF						4MBC						EMC					
		C1		C2		C3		C1		C2		C3		C1		C2		C3		C1		C2		C3	
		CA	IA	CA	IA	CA	IA	CA	IA	CA	IA	CA	IA	CA	IA	CA	IA	CA	IA	CA	IA	CA	IA	CA	IA
**mixture**	C1BPA+C1OXYB	0,732	1,533	0,575	1,79	0,252	1,173	0,872	1,69	0,692	1,34	0,531	1,06	0,786	1,537	0,677	1,250	0,720	1,211	0,538	1,049	0,580	1,056	0,620	1,038
	C1BPA+C2OXYB	1,265	2,457	0,569	1,67	0,255	1,168	0,921	1,65	0,734	1,36	0,555	1,10	0,717	1,298	0,735	1,293	0,764	1,265	0,633	1,142	0,668	1,159	0,701	1,153
	C1BPA+C3OXYB	1,207	2,055	0,457	1,17	0,286	1,186	1,025	1,61	0,739	1,24	0,630	1,17	0,781	1,245	0,739	1,179	0,790	1,230	0,711	1,129	0,757	1,190	0,765	1,186
	C2BPA+C1OXYB	1,128	2,163	0,365	1,61	0,265	1,268	0,903	1,56	0,569	1,35	0,568	1,13	0,777	1,363	0,608	1,279	0,775	1,259	0,661	1,155	0,571	1,186	0,748	1,207
	C2BPA+C2OXYB	1,099	1,997	0,594	1,69	0,274	1,256	0,979	1,62	0,790	1,39	0,598	1,17	0,773	1,293	0,764	1,266	0,765	1,223	0,686	1,142	0,727	1,183	0,797	1,265
	C2BPA+C3OXYB	1,051	1,716	0,491	1,22	0,302	1,232	1,030	1,54	0,768	1,24	0,666	1,21	0,806	1,224	0,763	1,166	0,773	1,168	0,834	1,261	0,840	1,263	0,839	1,260
	C3BPA+C1OXYB	0,906	1,531	0,552	1,43	0,287	1,204	0,869	1,35	0,735	1,22	0,611	1,14	0,763	1,200	0,715	1,128	0,735	1,134	0,726	1,138	0,767	1,191	0,741	1,138
	C3BPA+C2OXYB	0,925	1,531	0,585	1,45	0,297	1,199	0,905	1,38	0,792	1,30	0,618	1,13	0,740	1,143	0,726	1,126	0,744	1,139	0,759	1,168	0,799	1,221	0,817	1,243
	C3BPA+C3OXYB	1,084	1,679	0,539	1,22	0,321	1,183	0,994	1,43	0,788	1,22	0,668	1,16	0,774	1,127	0,755	1,111	0,765	1,123	0,827	1,198	0,837	1,214	0,852	1,244

Supplementary Table S2. MDR results of studies on impact of three component mixture on BPA, BPS, BPF, 4MBC and EMC toxicity (MDR values >2.0 exhibit antagonism, MDRs <0.5 show synergism, MDR values within 0.50–0.71 and 1.40–2.00 values mean, respectively, under- and overestimation of presented models; for values of particular concentrations C1, C2 and C3 of all analytes, please refer to subchapter 4.2. in the main text) (n=2)

		BPS						BPF						EMC					
		C1		C2		C3		C1		C2		C3		C1		C2		C3	
		CA	IA	CA	IA	CA	IA	CA	IA	CA	IA	CA	IA	CA	IA	CA	IA	CA	IA
**mixture**	C1BPA+C14MBC	1,240	2,583	0,562	1,79	0,337	1,621	0,861	1,64	0,659	1,27	0,581	1,18	0,554	1,062	0,663	1,203	0,759	1,263
	C1BPA+C24MBC	0,630	1,226	0,500	1,46	0,327	1,487	0,722	1,30	0,673	1,25	0,614	1,22	0,665	1,135	0,714	1,197	0,833	1,331
	C1BPA+C34MBC	0,926	1,620	0,762	1,92	0,359	1,425	0,726	1,20	0,781	1,35	0,659	1,23	0,717	1,104	0,795	1,230	0,835	1,270
	C2BPA+C14MBC	1,112	2,101	0,368	1,64	0,323	1,568	0,859	1,45	0,553	1,31	0,634	1,26	0,627	1,123	0,555	1,215	0,764	1,256
	C2BPA+C24MBC	0,922	1,683	0,512	1,45	0,310	1,416	0,799	1,33	0,713	1,26	0,639	1,25	0,712	1,173	0,768	1,256	0,840	1,330
	C2BPA+C34MBC	0,920	1,566	0,923	2,31	0,382	1,531	0,753	1,20	0,825	1,39	0,683	1,27	0,749	1,143	0,831	1,276	0,839	1,273
	C3BPA+C14MBC	0,987	1,647	0,523	1,35	0,321	1,360	0,860	1,31	0,748	1,23	0,665	1,23	0,689	1,142	0,742	1,225	0,829	1,328
	C3BPA+C24MBC	0,923	1,532	0,602	1,50	0,326	1,317	0,805	1,24	0,805	1,32	0,656	1,20	0,740	1,177	0,828	1,320	0,848	1,326
	C3BPA+C34MBC	0,904	1,455	0,621	1,42	0,378	1,381	0,769	1,16	0,825	1,32	0,690	1,22	0,780	1,182	0,843	1,287	0,830	1,258

Supplementary Table S3. MDR results of studies on impact of three component mixture on BPA, BPS, BPF, 4MBC and EMC toxicity (MDR values >2.0 exhibit antagonism, MDRs <0.5 show synergism, MDR values within 0.50–0.71 and 1.40–2.00 values mean, respectively, under- and overestimation of presented models; for values of particular concentrations C1, C2 and C3 of all analytes, please refer to subchapter 4.2. in the main text) (n=2)

		BPS							BPF					
		C1			C2		C3		C1		C2		C3	
		CA	IA	CA		IA	CA	IA	CA	IA	CA	IA	CA	IA
mixture	C1BPA+C1EMC	0,48	0,99	0,56		1,08	0,63	1,09	0,582	1,102	0,565	1,091	0,530	1,075
	C1BPA+C2EMC	0,58	1,85	0,34		1,01	0,42	1,06	0,669	1,186	0,611	1,128	0,565	1,124
	C1BPA+C3EMC	0,22	1,05	0,24		1,10	0,28	1,11	0,729	1,201	0,667	1,148	0,613	1,144
	C2BPA+C1EMC	0,572	1,08	0,633		1,14	0,678	1,15	0,694	1,166	0,483	1,138	0,567	1,129
	C2BPA+C2EMC	0,37	1,67	0,39		1,11	0,49	1,22	0,745	1,211	0,664	1,159	0,598	1,163
	C2BPA+C3EMC	0,23	1,14	0,25		1,15	0,301	1,21	0,776	1,224	0,750	1,256	0,637	1,180
	C3BPA+C1EMC	0,677	1,13	0,691		1,13	0,718	1,15	0,773	1,175	0,690	1,131	0,617	1,139
	C3BPA+C2EMC	0,531	1,37	0,448		1,11	0,523	1,20	0,781	1,178	0,714	1,159	0,634	1,156
	C3BPA+C3EMC	0,270	1,15	0,282		1,15	0,328	1,20	0,791	1,188	0,767	1,224	0,668	1,182

Supplementary Table S4. MDR results of studies on impact of three component mixture on BPA, BPS, BPF, 4MBC and EMC toxicity (MDR values >2.0 exhibit antagonism, MDRs <0.5 show synergism, MDR values within 0.50–0.71 and 1.40–2.00 values mean, respectively, under- and overestimation of presented models; for values of particular concentrations C1, C2 and C3 of all analytes, please refer to subchapter 4.2. in the main text) (n=2)

		BPF						4MBC						EMC					
		C1		C2		C3		C1		C2		C3		C1		C2		C3	
		CA	IA	CA	IA	CA	IA	CA	IA	CA	IA	CA	IA	CA	IA	CA	IA	CA	IA
**mixture**	C1BPS+C1OXYB	0,804	1,67	0,714	1,44	0,523	1,06	0,855	1,775	0,827	1,606	0,787	1,373	0,497	1,031	0,359	1,15	0,204	0,995
	C1BPS+C2OXYB	0,935	1,79	0,730	1,41	0,546	1,10	0,885	1,697	0,820	1,517	0,858	1,475	0,568	1,088	0,372	1,11	0,223	1,051
	C1BPS+C3OXYB	0,978	1,63	0,740	1,29	0,614	1,16	1,013	1,700	0,938	1,565	0,912	1,474	0,698	1,168	0,468	1,21	0,280	1,180
	C2BPS+C1OXYB	0,613	2,00	0,397	1,31	0,378	0,96	0,420	1,342	0,323	1,372	0,831	2,100	0,561	1,081	0,273	1,18	0,226	1,056
	C2BPS+C2OXYB	0,577	1,74	0,490	1,36	0,405	1,05	0,581	1,729	0,666	1,861	0,747	1,848	0,624	1,142	0,407	1,15	0,266	1,197
	C2BPS+C3OXYB	0,601	1,56	0,501	1,25	0,451	1,11	0,668	1,720	0,716	1,777	0,712	1,630	0,728	1,201	0,485	1,21	0,300	1,214
	C3BPS+C1OXYB	0,337	1,68	0,275	1,19	0,265	0,98	0,312	1,510	0,344	1,570	0,409	1,629	0,641	1,115	0,444	1,12	0,272	1,090
	C3BPS+C2OXYB	0,341	1,63	0,293	1,26	0,274	1,04	0,336	1,574	0,364	1,612	0,390	1,530	0,685	1,172	0,473	1,17	0,304	1,194
	C3BPS+C3OXYB	0,349	1,48	0,307	1,22	0,305	1,11	0,372	1,559	0,295	1,183	0,338	1,228	0,754	1,213	0,544	1,24	0,337	1,226

Supplementary Table S5. MDR results of studies on impact of three component mixture on BPA, BPS, BPF, 4MBC and EMC toxicity (MDR values >2.0 exhibit antagonism, MDRs <0.5 show synergism, MDR values within 0.50–0.71 and 1.40–2.00 values mean, respectively, under- and overestimation of presented models; for values of particular concentrations C1, C2 and C3 of all analytes, please refer to subchapter 4.2. in the main text) (n=2)

		EMC					
		C1		C2		C3	
		CA	IA	CA	IA	CA	IA
mixture	C1BPS+C14MBC	0,517	1,062	0,587	1,12	0,675	1,166
	C1BPS+C24MBC	0,569	1,093	0,630	1,16	0,661	1,134
	C1BPS+C34MBC	0,685	1,189	0,721	1,23	0,765	1,263
	C2BPS+C14MBC	0,312	1,017	0,247	1,08	0,442	1,128
	C2BPS+C24MBC	0,372	1,108	0,408	1,14	0,499	1,233
	C2BPS+C34MBC	0,492	1,258	0,542	1,35	0,593	1,356
	C3BPS+C14MBC	0,222	1,112	0,257	1,22	0,300	1,219
	C3BPS+C24MBC	0,249	1,169	0,275	1,23	0,314	1,231
	C3BPS+C34MBC	0,311	1,266	0,350	1,38	0,376	1,343

Supplementary Table S6. MDR results of studies on impact of three component mixture on BPS, BPF and EMC toxicity (MDR values >2.0 exhibit antagonism, MDRs <0.5 show synergism, MDR values within 0.50–0.71 and 1.40–2.00 values mean, respectively, under- and overestimation of presented models; for values of particular concentrations C1, C2 and C3 of all analytes, please refer to subchapter 4.2. in the main text)(n=2)

		BPF					
		C1		C2		C3	
		CA	IA	CA	IA	CA	IA
mixture	C1BPS+C1EMC	0,537	1,098	0,529	1,07	0,528	1,083
	C1BPS+C2EMC	0,652	1,233	0,575	1,11	0,554	1,119
	C1BPS+C3EMC	0,705	1,216	0,654	1,16	0,596	1,127
	C2BPS+C1EMC	0,311	1,030	0,295	1,00	0,381	1,003
	C2BPS+C2EMC	0,380	1,150	0,379	1,06	0,403	1,055
	C2BPS+C3EMC	0,450	1,159	0,504	1,24	0,449	1,076
	C3BPS+C1EMC	0,215	1,101	0,219	0,98	0,255	0,978
	C3BPS+C2EMC	0,232	1,121	0,244	1,06	0,269	1,037
	C3BPS+C3EMC	0,276	1,140	0,323	1,23	0,305	1,070

Supplementary Table S7. MDR results of studies on impact of three component mixture on BPF, 4MBC, BPS, OXB and EMC toxicity (MDR values >2.0 exhibit antagonism, MDRs <0.5 show synergism, MDR values within 0.50–0.71 and 1.40–2.00 values mean, respectively, under- and overestimation of presented models; for values of particular concentrations C1, C2 and C3 of all analytes, please refer to subchapter 4.2. in the main text) (n=2)

		BPS						EMC						OXYB					
		C1		C2		C3		C1		C2		C3		C1		C2		C3	
		CA	IA	CA	IA	CA	IA	CA	IA	CA	IA	CA	IA	CA	IA	CA	IA	CA	IA
**mixture**	C1BPF+C14MBC	0,710	1,455	0,402	1,34	0,262	1,334	0,798	1,46	0,843	1,45	0,911	1,48	0,578	1,09	0,654	1,14	0,766	1,18
	C1BPF+C24MBC	0,656	1,258	0,396	1,19	0,252	1,195	0,843	1,48	0,871	1,47	0,952	1,54	0,625	1,12	0,676	1,16	0,761	1,18
	C1BPF+C34MBC	0,667	1,156	0,457	1,18	0,274	1,128	0,933	1,52	0,935	1,50	0,967	1,52	0,707	1,17	0,727	1,18	0,795	1,21
	C2BPF+C14MBC	0,599	1,205	0,340	1,15	0,239	1,061	0,665	1,27	0,580	1,37	0,685	1,17	0,533	1,03	0,588	1,08	0,721	1,19
	C2BPF+C24MBC	0,603	1,163	0,398	1,10	0,305	1,299	0,598	1,09	0,667	1,19	0,732	1,24	0,451	1,08	0,620	1,11	0,732	1,21
	C2BPF+C34MBC	0,782	1,389	0,535	1,32	0,328	1,246	0,688	1,18	0,726	1,23	0,771	1,27	0,643	1,11	0,706	1,20	0,773	1,25
	C3BPF+C14MBC	0,559	1,141	0,402	1,05	0,264	1,003	0,521	1,06	0,552	1,10	0,596	1,11	0,502	1,02	0,541	1,07	0,614	1,14
	C3BPF+C24MBC	0,569	1,144	0,424	1,09	0,279	1,048	0,542	1,08	0,586	1,15	0,641	1,19	0,541	1,07	0,554	1,08	0,623	1,15
	C3BPF+C34MBC	0,645	1,220	0,463	1,11	0,320	1,115	0,608	1,14	0,661	1,23	0,691	1,24	0,595	1,11	0,605	1,12	0,666	1,18

Supplementary Table S8. MDR results of studies on impact of three component mixture on BPA, BPS, BPF, 4MBC and EMC toxicity (MDR values >2.0 exhibit antagonism, MDRs <0.5 show synergism, MDR values within 0.50–0.71 and 1.40–2.00 values mean, respectively, under- and overestimation of presented models; for values of particular concentrations C1, C2 and C3 of all analytes, please refer to subchapter 4.2. in the main text) (n=2)

		EMC					
		C1		C2		C3	
		CA	IA	CA	IA	CA	IA
**mixture**	C1BPF+C1 OXYB	0,584	1,098	0,647	1,14	0,714	1,172
	C1BPF+C2 OXYB	0,659	1,148	0,697	1,17	0,734	1,182
	C1BPF+C3 OXYB	0,770	1,184	0,780	1,19	0,801	1,212
	C2BPF+C1 OXYB	0,537	1,033	0,460	1,09	0,648	1,112
	C2BPF+C2 OXYB	0,591	1,082	0,634	1,13	0,712	1,205
	C2BPF+C3 OXYB	0,724	1,196	0,747	1,22	0,778	1,248
	C3BPF+C1 OXYB	0,504	1,023	0,551	1,09	0,598	1,116
	C3BPF+C2 OXYB	0,543	1,079	0,563	1,09	0,608	1,128
	C3BPF+C3 OXYB	0,616	1,139	0,632	1,16	0,669	1,186

Supplementary Table S9. MDR results of studies on impact of three component mixture on OXYB, 4MBC and EMC toxicity (MDR values >2.0 exhibit antagonism, MDRs <0.5 show synergism, MDR values within 0.50–0.71 and 1.40–2.00 values mean, respectively, under- and overestimation of presented models; for values of particular concentrations C1, C2 and C3 of all analytes, please refer to subchapter 4.2. in the main text) (n=2)

		EMC					
		C1		C2		C3	
		CA	IA	CA	IA	CA	IA
**mixture**	C14MBC+C1 OXYB	0,670	1,275	0,712	1,27	0,774	1,279
	C14MBC+C2 OXYB	0,779	1,372	0,781	1,33	0,846	1,372
	C14MBC+C3 OXYB	0,836	1,299	0,819	1,26	0,931	1,421
	C24MBC+C1 OXYB	0,781	1,410	0,702	1,53	0,916	1,508
	C24MBC+C2 OXYB	0,764	1,315	1,005	1,69	1,126	1,827
	C24MBC+C3 OXYB	1,062	1,656	1,070	1,66	1,075	1,651
	C34MBC+C1 OXYB	0,886	1,468	0,976	1,60	0,981	1,568
	C34MBC+C2 OXYB	0,904	1,472	0,909	1,47	0,984	1,561
	C34MBC+C3 OXYB	0,999	1,529	0,927	1,42	0,905	1,379
